# Supplementary figures and images for: Clinical characteristics of patients with confirmed and asymptomatic SARS-CoV-2 infection in China
Source: PLoS One. 2022 Aug 23;17(8):e0273150. doi: 10.1371/journal.pone.0273150 (PMC9397853; doi:10.1371/journal.pone.0273150)

**S1 Fig. Comparison of CT scans before and after expert annotation**
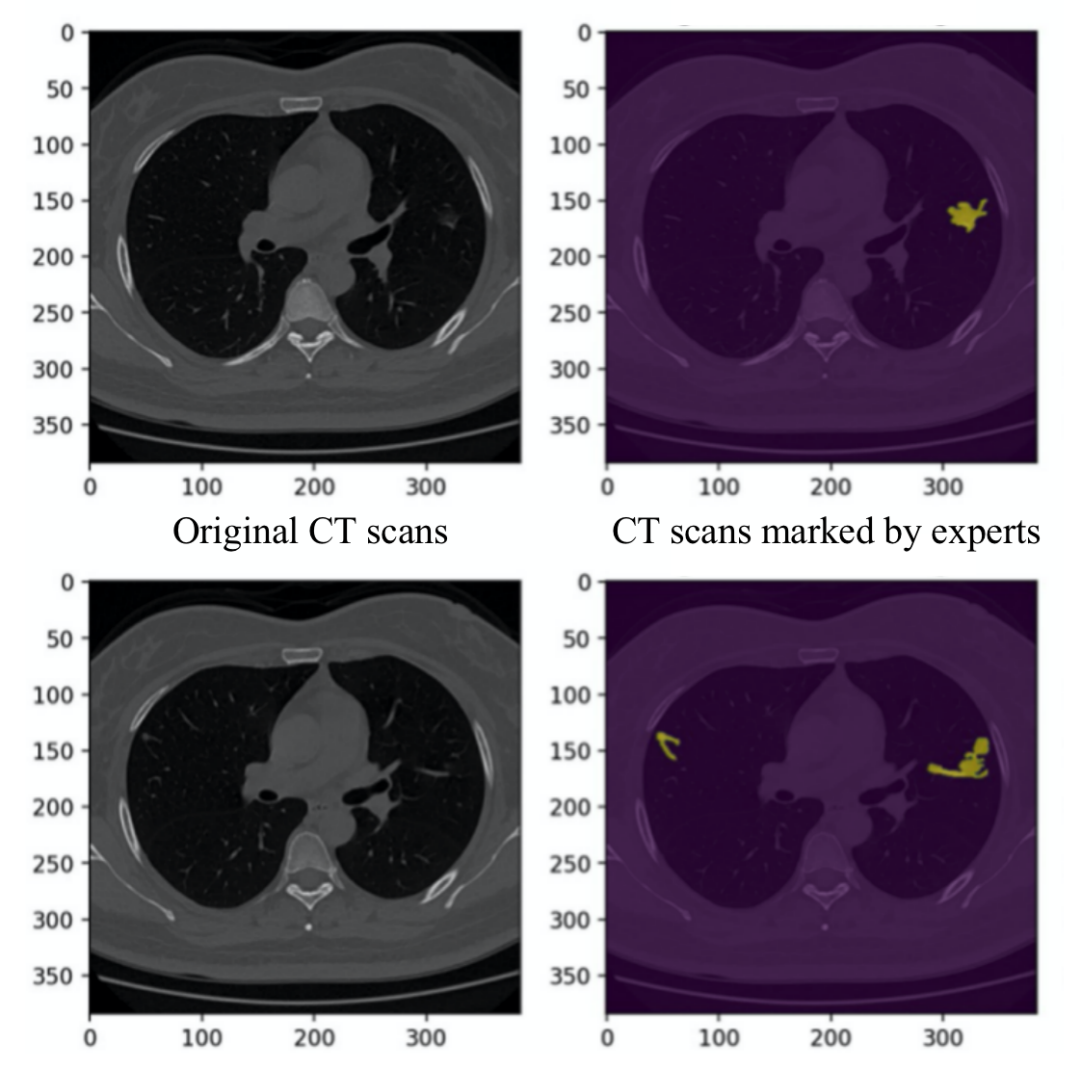

Supplement: S1 Fig — (DOCX) [file pone.0273150.s001.docx]

**S2 Fig. Chest CT scans from six patients with different lesion-ratio**
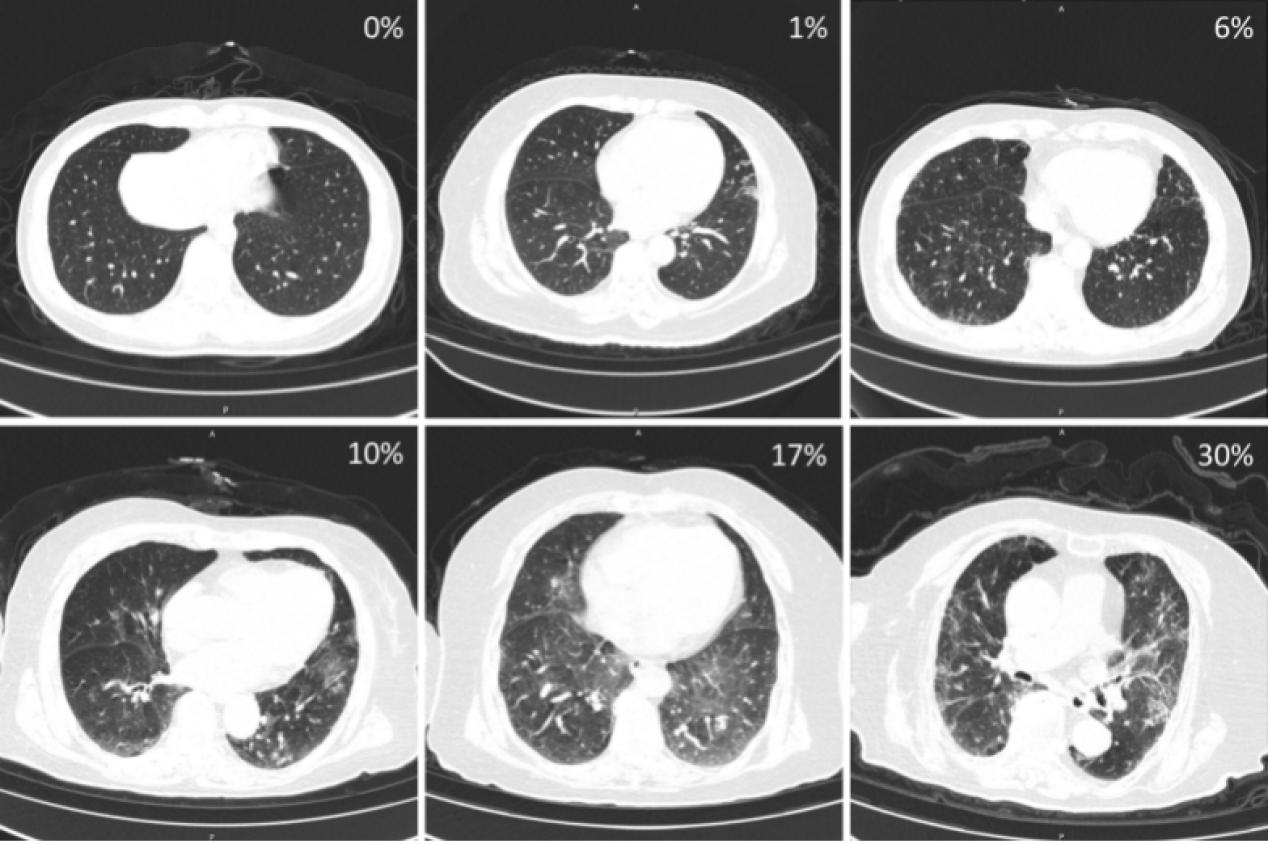

Supplement: S2 Fig — (DOCX) [file pone.0273150.s002.docx]

**S4 Fig. ROC curve of the multivariable logistic regression model**


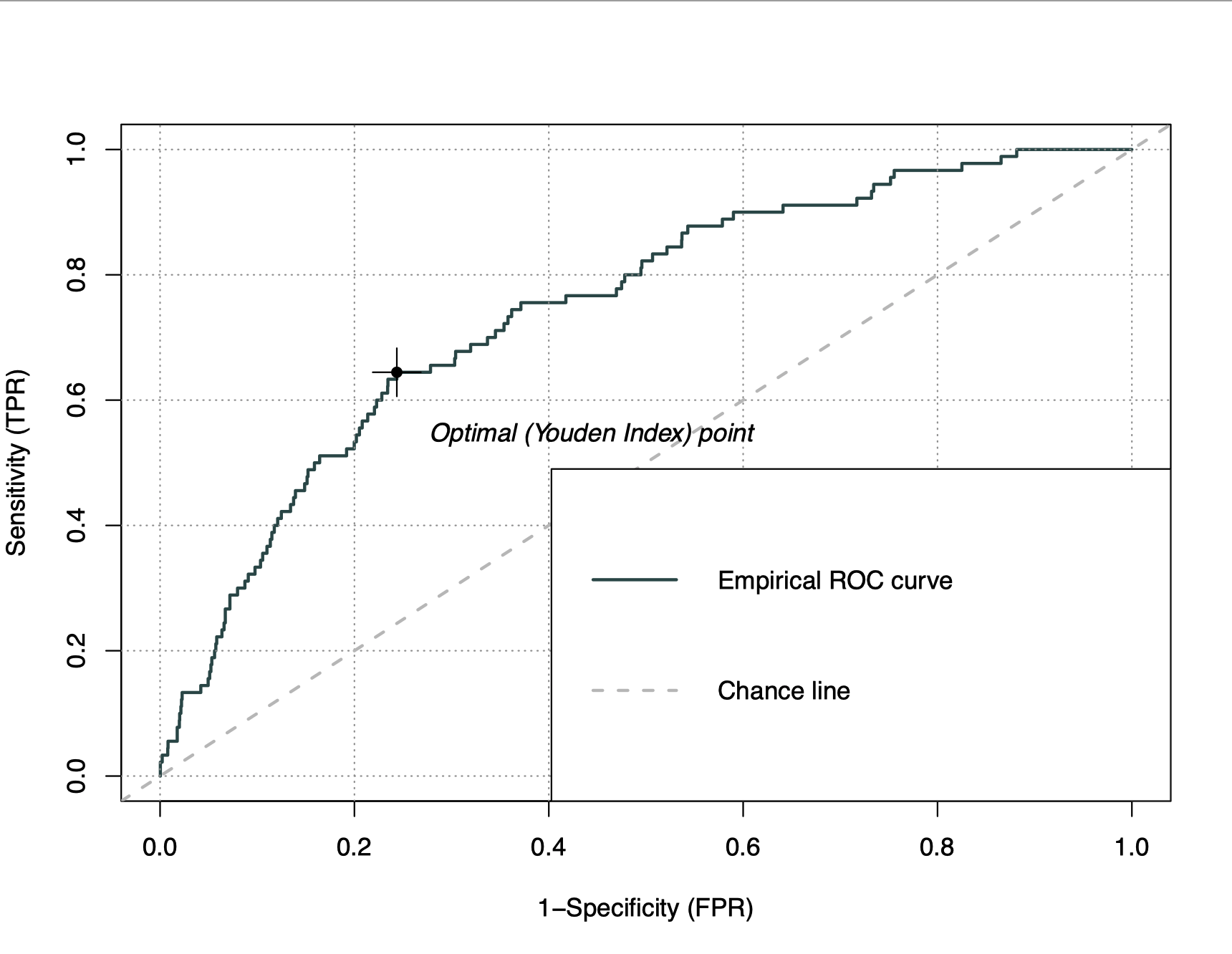

Supplement: S4 Fig — (DOCX) [file pone.0273150.s004.docx]
