# Supplementary material for: Clinical characteristics of patients with confirmed and asymptomatic SARS-CoV-2 infection in China
Source: PLoS One. 2022 Aug 23;17(8):e0273150. doi: 10.1371/journal.pone.0273150 (PMC9397853; doi:10.1371/journal.pone.0273150)
Supplement: S3 Fig — (a) LASSO coefficient profiles of the 36 baseline features. (b) Tuning parameter (λ) selection in the LASSO model used 10-fold cross-validation via minimum criteria. (DOCX) [file pone.0273150.s003.docx]

**S3 Fig. The least absolute shrinkage and selection operator (LASSO) binary logistic regression model (a) LASSO coefficient profiles of the 36 baseline features. (b) Tuning parameter (λ) selection in the LASSO model used 10-fold cross-validation via minimum criteria**


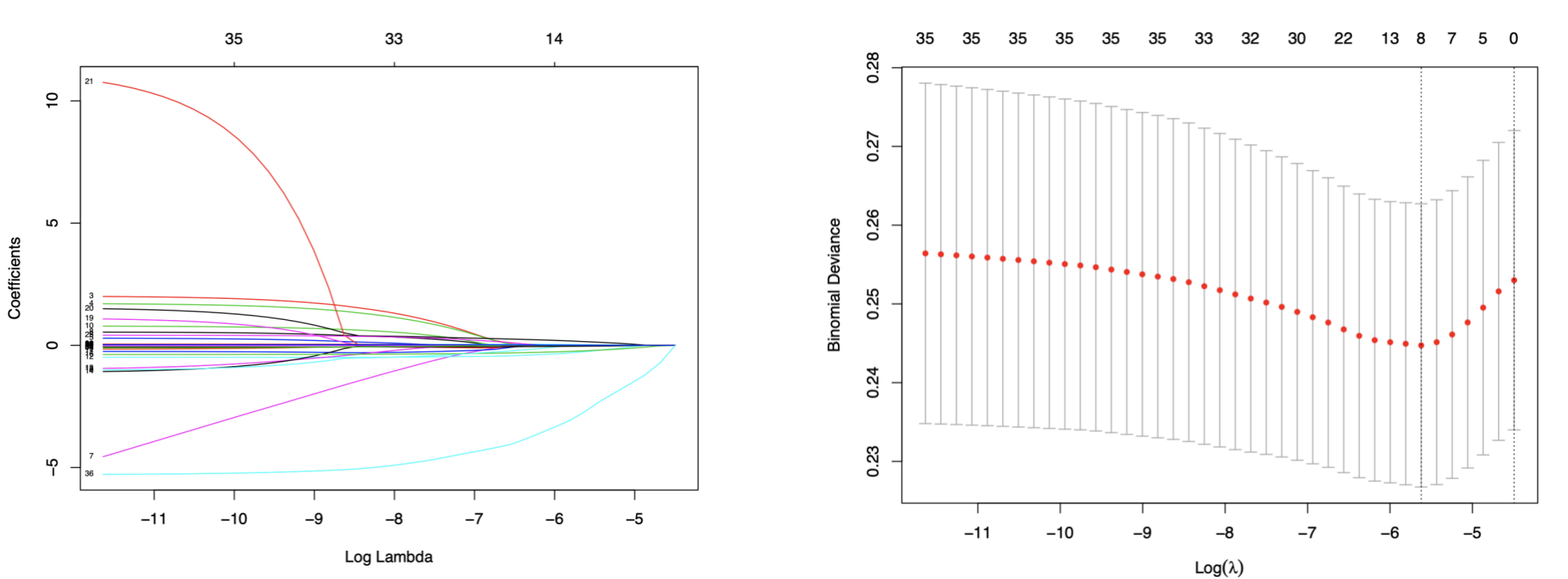


1. **(b)**
