# Supplementary material for: Clinical characteristics of patients with confirmed and asymptomatic SARS-CoV-2 infection in China
Source: PLoS One. 2022 Aug 23;17(8):e0273150. doi: 10.1371/journal.pone.0273150 (PMC9397853; doi:10.1371/journal.pone.0273150)
Supplement: Attachment — Submitted filename: Response to Reviewers.docx [file pone.0273150.s005.docx]

# Response to Reviewer #1:

**Comments of reviewer:**

Vancouver style reference format to be used for listing the references in the article. Subsequently, alterations to be made in Reference 1, 7,8 and 14.

Some formatting required in the participant section in the Abstract to ensure that only the necessary subject headings are highlighted.

**Response to reviewer**

We are glad to receive your further feedback. We have made corresponding changes according to your comments.

Specifically, we have updated the Vancouver format that meets the requirements of the "PLOS ONE" format, while updating some real-time statistics and replacing some more appropriate references (reference 1,7,8 and 14). Moreover, we highlight the important part in the participant section in the Abstract to ensure that only the necessary subject headings are highlighted.
